# Ultrasound-guided stellate ganglion blockade – patient positioning is everything: a case report demonstrating the efficacy of a modified out-of-plane approach

**DOI:** 10.3389/fnins.2023.1288484

**Published:** 2024-01-16

**Authors:** Ke Mo, Liang Qian, Jingling Tian, Junling Liao, Fang Tan, Weirong Kong, Xianjun Yu, Xinjin Chi

**Affiliations:** Department of Anesthesiology, The Seventh Affiliated Hospital of Sun Yat-sen University, Shenzhen, Guangdong, China

**Keywords:** insomnia, ultrasound guidance, stellate ganglion block, lateral paravein approach, out-of-plane

## Abstract

**Background:**

Insomnia has become increasingly prevalent in modern society and is notoriously difficult to treat. Many patients exhibit a poor response to pharmacological interventions. Stellate ganglion block (SGB) has emerged as an effective method for managing insomnia; however, its efficacy may be compromised in some patients, primarily due to a variant vertebral artery anatomy.

**Case presentation:**

This case report describes a patient with severe insomnia accompanied by anxiety. Through cervical ultrasound scanning, we identified richly branched cervical arteries at the C6−C7 segment of the vertebral artery, along with anatomical variations, which could pose a heightened risk for the traditional SGB procedure. Therefore, after carefully adjusting the patient’s positioning, we proceeded with ultrasound-guided SGB using a lateral paravein out-of-plane approach. Clinical signs of successful insomnia symptoms alleviation were consistently observed after each block utilizing this alternative technique multiple times in a single patient.

**Conclusion:**

Our report reveals a new lateral paravein out-of-plane approach for ultrasound-guided SGB to treat insomnia, which might be considered an alternative method. More studies should be carried out to confirm the efficacy of this new approach.

## Introduction

Insomnia, a pervasive sleep disorder characterized by difficulties in initiating or maintaining sleep, presents significant challenges for effective treatment (Leger and Poursain, 2005). Traditional pharmacological approaches often yield suboptimal results and are burdened with potential side effects and concerns regarding dependency. In recent years, several alternative interventions, such as stellate ganglion block (SGB), have emerged as promising therapeutic options for insomnia (Haest et al., 2012).

The stellate ganglion, located in the neck region, plays a vital role in regulating the activity of the sympathetic nervous system (Erickson and Hogan, 1993). By modulating or blocking the function of the stellate ganglion, SGB aims to restore autonomic balance and alleviate the symptoms associated with insomnia (Haest et al., 2012; Hanling et al., 2016; Gu et al., 2022). However, the presence of an atypical vertebral artery anatomy can complicate the procedure, thereby making it more challenging (Bruneau et al., 2006). These anatomical variations can increase the risk of complications occurring during SGB, including potential nerve damage or vascular injury (Takanami et al., 2009). Hence, it is imperative to discern and navigate these intricate anatomical complexities to ensure the safety and effectiveness of the SGB procedure.

In this report, we present a noteworthy case of a patient suffering from severe insomnia with a variant vertebral artery anatomy. In the case of this patient, it was considered that the conventional lateral in-plane approach for the SGB procedure might pose a heightened risk. Consequently, we utilized a lateral paravein out-of-plane approach with adjusting the patient’s position accordingly, and we were then able to successfully perform the SGB procedure. This intervention effectively mitigated the sleep symptoms encountered by the patient.

## Case report

### Medical history

A 56-year-old female presented with a primary complaint of persistent insomnia and anxiety over the past 3°years, which had progressively worsened over the last 3°months. The patient reported chronic psychological distress arising from familial conflicts, resulting in difficulties falling asleep. Typically, she reported experiencing a delay of 1−2 h before falling asleep on almost a daily basis. Her sleep was described as shallow without any instances of snoring or choking, but frequently interrupted by 3−4 awakenings throughout the night. These interruptions further hindered her ability to return to sleep, leading to fatigue, daytime drowsiness, and mood fluctuations. Although she reported occasionally relying on medication to get to sleep, such as diazepam, these interventions had not significantly alleviated her symptoms. Previously, the patient had underwent SGB treatment at another tertiary hospital; unfortunately, this resulted in the development of a neck hematoma and hoarseness of her voice. Since experiencing these adverse effects, her condition had gradually deteriorated.

Based on the patient’s diagnosis and treatment records regarding previous insomnia treatments received at other medical facilities, we performed routine physical examination (e.g., maxillofacial and throat examination, cardiopulmonary auscultation, electrocardiogram, and blood pressure) and laboratory tests (routine blood tests, coagulation function, liver and kidney function, and blood sugar tests) when the patient was first seen. These examinations did not reveal any significant abnormalities. Simultaneously, we assessed the patient’s Sleep Apnea Scale (SAS) scores and Insomnia Severity Index (ISI) scores. The results revealed that the patient obtained an SAS score of 64 and an ISI score of 15, thus confirming the presence of both insomnia and anxiety. Based on these findings, the patient was diagnosed with chronic insomnia using the Guidelines for Diagnosis and Treatment of Insomnia in China (Chinese Sleep Research Society, 2017). With the patient’s informed consent and explicit agreement to proceed with treatment, she underwent ultrasound-guided SGB using the lateral paravein out-of-plane approach at our medical facility.

### Treatment plan

**FIGURE 1:**
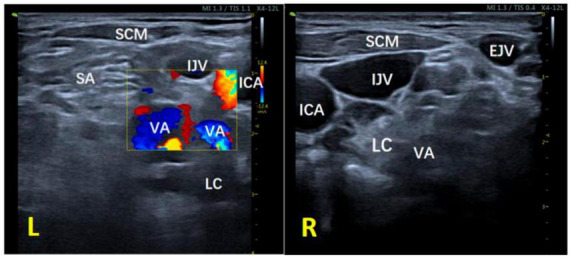
Ultrasound image of the neck at the C6–C7 segment of the patient. L, left; R, right; IJV, internal jugular vein; EJV, external jugular vein; ICA, internal carotid artery; VA, vertebral artery; LC, longus colli muscle; SA, anterior oblique muscle; SCM, sternocleidomastoid muscle; TP, transverse process.

Initially, the neck vessels were examined by an ultrasound scan. The results indicated that the left vertebral artery and its branches covered a significant portion of the longus colli muscle at the C6−C7 level, while the right vertebral artery ran outside the longus colli muscle at the same level (Figure 1). Consequently, it was determined that a bilateral paracarotid approach would not be suitable for this patient. Instead, we proposed a new method for SGB called the lateral paravein out-of-plane approach. This approach is similar to a recently reported ultrasound-guided SGB technique that employs the lateral out-of-plane approach but eliminates the need to puncture the internal jugular vein, which is a useful benefit, because following the puncture of the internal jugular vein, some patients may experience local hematoma, venous inflammation, venous thrombosis, pneumothorax, and other complications. Although these complications are not frequently encountered during the procedure, attempts should be undertaken to mitigate their incidence (Kim et al., 2017).

**FIGURE 2:**
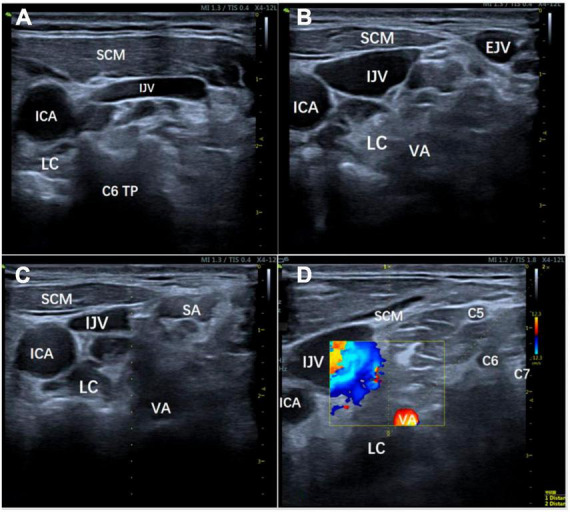
Position adjustment process. **(A)** The ultrasound image clearly displays the transverse process of C6, as well as its anterior and posterior lymph nodes, along with the anatomical relationship between the sternocleidomastoid muscle, internal jugular vein, and carotid artery in a supine position; **(B)** To maximize the leftward extension of the internal jugular vein, the patient’s head was rotated 60° to the left or positioned with the head elevated 30° and feet lowered; **(C, D)** The patient was instructed to slightly open her mouth, relax the anterior neck muscles, and stay steady to allow for the planning of the puncture path using color Doppler and ultrasound guidance to avoid important structures. IJV, internal jugular vein; EJV, external jugular vein; ICA, internal carotid artery; VA, vertebral artery; LC, longus colli muscle; SA, scalenus anterior muscle; SCM, sternocleidomastoid muscle; TP, transverse process.

Specifically, the patient was positioned in a supine position with her head tilted backward. The long axis of the ultrasound probe was aligned parallel to the plane of the cricoid cartilage, forming a 45° angle with the sagittal plane of the neck. Moving from the medial side, the probe was used to scan beyond the lateral border of the sternocleidomastoid muscle. The ultrasound image clearly displayed the transverse process of C6, as well as its anterior and posterior lymph nodes, along with the anatomical relationship between the sternocleidomastoid muscle, internal jugular vein, and carotid artery (Figure 2A). However, the presence of a prominent anterior tubercle on the C6 transverse process hindered the puncture. To overcome this hurdle, the ultrasound probe was slightly shifted toward the direction of C7 until the anterior tubercle was no longer visible on the screen. Subsequently, the patient was asked to rotate her head 60° to the left, which maximally extended the internal jugular vein toward the left. Although not an issue in the presented case, in cases where the internal jugular vein remains filled to a significant extent, it may be beneficial to contemplate assuming a 30° head-up, feet-down posture, while also inclining the patient’s head toward the opposite side to elongate the sternocleidomastoid muscle (Figure 2B).

**FIGURE 3:**
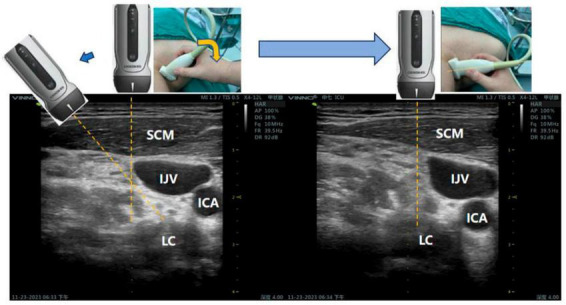
Ultrasonic probe adjustment method: The dashed yellow line represents the median line of the ultrasound probe and served as a guide for the puncture. IJV, internal jugular vein; ICA, internal carotid artery; LC, longus colli muscle; SCM, sternocleidomastoid muscle.

The patient was instructed to open her mouth slightly, relax the anterior neck muscles, and to stay steady to allow for the planning of the puncture path, which was done using color Doppler and ultrasound guidance, avoiding important structures, such as the vertebral artery, internal jugular vein, and cervical nerves (Figures 2C, D). The crucial step in enhancing the lateral paravein out-of-plane approach for SGB involves conducting puncture guidance along the median line of the linear array probe. By rotating the probe adjacent to the patient’s internal jugular vein, with the patient’s spine serving as the axis, the most optimal route for administering the SGB can be identified. It is essential to record the requisite distance for the puncturing and subsequently implement the puncture on the exterior of the internal jugular vein while following the guidance delineated along the median line (Figure 3).

**FIGURE 4:**
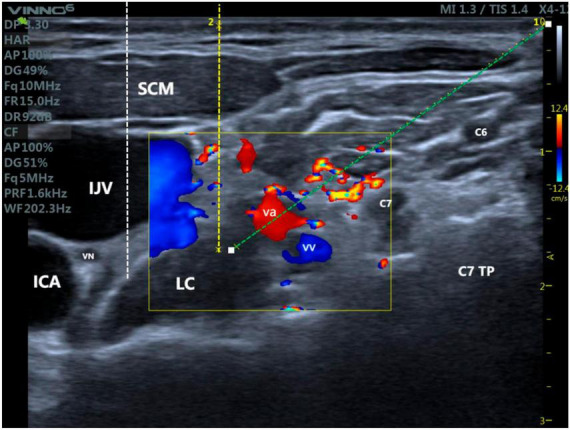
Differences between ultrasound-guided stellate ganglion blocks with three different access routes: Yellow dotted line: Stellate ganglion block with an out-of-plane approach adjacent to the internal jugular vein; White dotted line: Stellate ganglion block with an out-of-plane approach involving puncturing the internal jugular vein; Green dotted line: Lateral in-plane approach for stellate ganglion block. IJV, internal jugular vein; EJV, external jugular vein; ICA, internal carotid artery; VA, vertebral artery; LC, longus colli muscle; SA, scalenus anterior muscle; SCM, sternocleidomastoid muscle; TP, transverse process; VN, vagus nerve.

The puncture pathway can be simulated by opening the guide wire. Distance 1 corresponds to an out-of-plane approach, which is adjacent to the outer aspect of the internal jugular vein. The simulated puncture distance for this approach in the present case was 2.59 cm (Supplementary Figure 1). Distance 2 represents the in-plane approach from the lateral aspect, with a simulated puncture distance of 3.63 cm, which posed a higher risk here due to the presence of the carotid and vertebral arteries along the puncture trajectory. It is advisable to compress the internal jugular vein as much as possible before performing the puncture. By using the out-of-plane approach, the needle was advanced along the puncture guide wire, and a saline test was performed during the procedure to verify the position of the needle tip (Figure 4). Once the needle position was confirmed, 5 mL of 1% lidocaine was injected into the surface of the longus colli muscle and the deep aspect of the prevertebral fascia in order to complete the blockade. The success of the blockade was determined by observing the presence of Horner’s syndrome (drooping eyelid, constricted pupil, and sunken eye), as well as flushing of the conjunctiva and skin on the same side, blockage of the nose, and a lack of sweating on the blocked side.

Following the procedure, the patient was subjected to a 30-minute observation period in the hospital. We administered SGB therapy to the patient every other day, along with 12-day pyrazolone treatment (starting initially with a dosage of 10 mg and then reducing this to 5 mg on the fourth day). After eight sessions of SGB therapy, on the 15th day, the patient’s initial ISI score of 15 had decreased to 4, and their PSQI score had improved from 17 (indicating poor sleep quality, assessed on the third day) to 8 (indicating good sleep quality). Additionally, there was also a notable improvement in the SAS standard score, reducing from 64 (moderate anxiety) to 56 (mild anxiety). Importantly, the patient did not report experiencing any discomfort throughout the entire treatment process. Furthermore, there were no occurrences of SGB-related complications, such as infection or bleeding, and no signs of nerve damage were observed.

## Discussion

Since the first appearance of ultrasound-guided SGB, it has been extensively utilized in the field of non-anesthesiology sedation, exhibiting remarkable therapeutic efficacy in various conditions, including pain management, sleep disturbances, immunological disorders, irregular heart rhythms, mental health ailments, and endocrine dysfunctions (Deng et al., 2023). Compared to the traditional blind technique, ultrasound-guided SGB enhances the precision of nerve blockade while reducing the use of anesthesia. Furthermore, the use of ultrasound enables the direct visualization of vascular structures (internal jugular vein, inferior thyroid artery, carotid artery) as well as soft tissues (esophagus, thyroid, and nerve roots), thereby enhancing the safety of SGB. The scientific literature offers many insights into different puncture approaches for SGB, which include the paratracheal, trans-thyroidal, lateral paracarotid out-of-plane, and lateral in-plane approaches (Elias, 2000). Currently, the preferred method for ultrasound-guided SGB involves the lateral in-plane approach at the C6 or C7 level, while the out-of-plane approach is less favored (Kim et al., 2017). This preference arises from the challenges associated with poor visualization of the needle tip and the higher occurrence of complications linked to the out-of-plane technique (Schafhalter-Zoppoth et al., 2004). Although conclusive evidence favoring one approach over the other is lacking, the operator’s skill significantly influences needle visibility, thus leading to a greater reliance on the in-plane approach (Jung et al., 2011).

Our study proposes a highly novel method for SGB using a lateral paravein out-of-plane approach, thereby minimizing the risk of needle-induced injury to the internal jugular vein. Additionally, this new approach simplifies the procedure, thereby reducing the complexity associated with skill acquisition and potential complications. In the case presented, the patient exhibited an anatomical anomaly involving the vertebral artery. Specifically, on the left side, the vertebral artery at the C6−C7 level completely enveloped the surface of the longus colli muscle. Conversely, on the right side, the vertebral artery at the same level was situated above the external longus colli muscle. Further examination revealed the presence of the cervical nerve and external jugular vein along the intended puncture route. It has been reported that navigating through these critical structures presents significant challenges when employing a lateral in-plane approach (Jadon, 2011).

A previous study introduced an out-of-plane approach for SGB that involved puncturing the internal jugular vein next to the internal carotid artery to reach the surface of the longus colli muscle (Kim et al., 2017). In another study, skilled doctors were able to complete this technique in just 1−2 min. In comparison, the mainstream C6 modified in-plane SGB had an average operating time of 5.1 ± 0.6 min, while the C7 in-plane SGB took 8.2 ± 1.4 min (Rae Olmsted et al., 2019). However, it is important to note that this alternative approach requires piercing the internal jugular vein, which increases the risk of complications, such as hoarseness of the voice, compared to the lateral in-plane approach (Higa et al., 2006). Factors like potential injury to the internal jugular vein, hoarseness caused by vagus nerve block, and difficulties in localizing the needle tip limit the application of out-of-plane techniques in SGB.

The proposed technique for this patient, namely ultrasound-guided SGB using the lateral paravein out-of-plane approach, offered the shortest puncture route directly to the surface of the longus colli muscle. It only required adjusting the patient position, applying pressure to the probe, and rotating the probe along the spinal axis to achieve the best outcome. Additionally, the puncture route avoided the vertebral artery and vein, external jugular vein, and transverse process. With a puncture time of approximately 1.74 ± 0.19 min, our technique was quicker than the in-plane approach, and comparable to SGB with the out-of-plane approach, which involves piercing the internal jugular vein. Meanwhile, the drug injection location in our SGB approach aligns with that of the lateral in-plane approach, significantly reducing the risk of vagus nerve injury and hematoma formation from an anatomical perspective. Based on our experience of performing SGB on this patient eight times, we did not observe any complications, such as hoarseness, abnormal sensations, or hematoma. Within 3 min after the block, the patient developed Horner’s syndrome. The mean operation time was 1.74 min, with a 100% success rate.

During the treatment period, the patient reported an improvement in the symptoms of difficulty in falling asleep after receiving the fourth session of SGB therapy on the 7th day. Further, the patient’s PSQI score decreased from 17 on the third day to 11, the ISI score decreased to 7, and the SAS score decreased to 60. Consequently, pyrazolone was discontinued on the 10th day. Except for an additional dose of pyrazolone taken on the 12th day due to special reasons, the patient reported being able to fall asleep within 30 min and perceived an improvement in sleep quality compared to before. An assessment was conducted on the 15th day, revealing a PSQI score of 8, an ISI score of 4, and an SAS score of 56 for the patient. Both objective evaluations and the patient’s subjective experience indicated improvements in sleep quality and anxiety levels compared to before the treatment.

Given the positive outcome of this case and our prior knowledge, we have endeavored to employ this approach in patients possessing unaltered anatomical structures. As soon as a SGB procedure becomes arduous to finalize, our strategy entails an immediate transition to the traditional SGB technique. The findings to date indicate that the majority of patients with an intact anatomy could safely and expeditiously undergo SGB using this approach, without any accompanying complications. Despite undergoing a series of positional adjustments, some patients continued to experience significant venous congestion in the internal jugular vein, making it difficult to achieve a satisfactory out-of-plane puncture pathway. Additionally, we identified a case where a cyst was present adjacent to the internal jugular vein during ultrasound examination. For these patients, utilizing the current conventional lateral in-plane approach remains a safer and more effective choice.

Given the advantages discussed above, we regard ultrasound-guided SGB using the lateral paravein out-of-plane approach as a valuable clinical technique. It complements the in-plane method and effectively mitigates the risks associated with punctures. Furthermore, this technique offers theoretical validation for the extensive application of SGB techniques in the treatment of diverse ailments, thereby benefiting a broader patient demographic.

## Informed consent

Written informed consent to participate in this study was provided by the participant. Also, written informed consent was obtained from the individual for the publication of any potentially identifiable images or data included in this article.

## Data availability statement

The original contributions presented in this study are included in this article/Supplementary material, further inquiries can be directed to the corresponding author.

## Ethics statement

Ethical approval was not required for the studies involving humans because the case report is not applicable for ethical approval. The studies were conducted in accordance with the local legislation and institutional requirements. The participants provided their written informed consent to participate in this study. Written informed consent was obtained from the individual(s) for the publication of any potentially identifiable images or data included in this article.

## Author contributions

KM: Writing – original draft. LQ: Writing – original draft. JT: Supervision, Writing – review & editing. JL: Visualization, Writing – review & editing. FT: Validation, Writing – review & editing. WK: Investigation, Writing – review & editing. XY: Data curation, Writing – review & editing. XC: Writing – review & editing.

## Supplementary material

Supplementary Figure 1 Ultrasonographic image of the needle paths for stellate ganglion block using the lateral paravein out-of-plane approach. The yellow dashed line in the actual diagram shows the out-of-plane and in-plane approaches to the internal jugular vein, corresponding to Distance 1 and Distance 2 in the ultrasound image, respectively.
