# Supplementary figures and images for: Ultrasound-guided stellate ganglion blockade – patient positioning is everything: a case report demonstrating the efficacy of a modified out-of-plane approach
Source: Front Neurosci. 2024 Jan 16;17:1288484. doi: 10.3389/fnins.2023.1288484 (PMC10825014; doi:10.3389/fnins.2023.1288484)

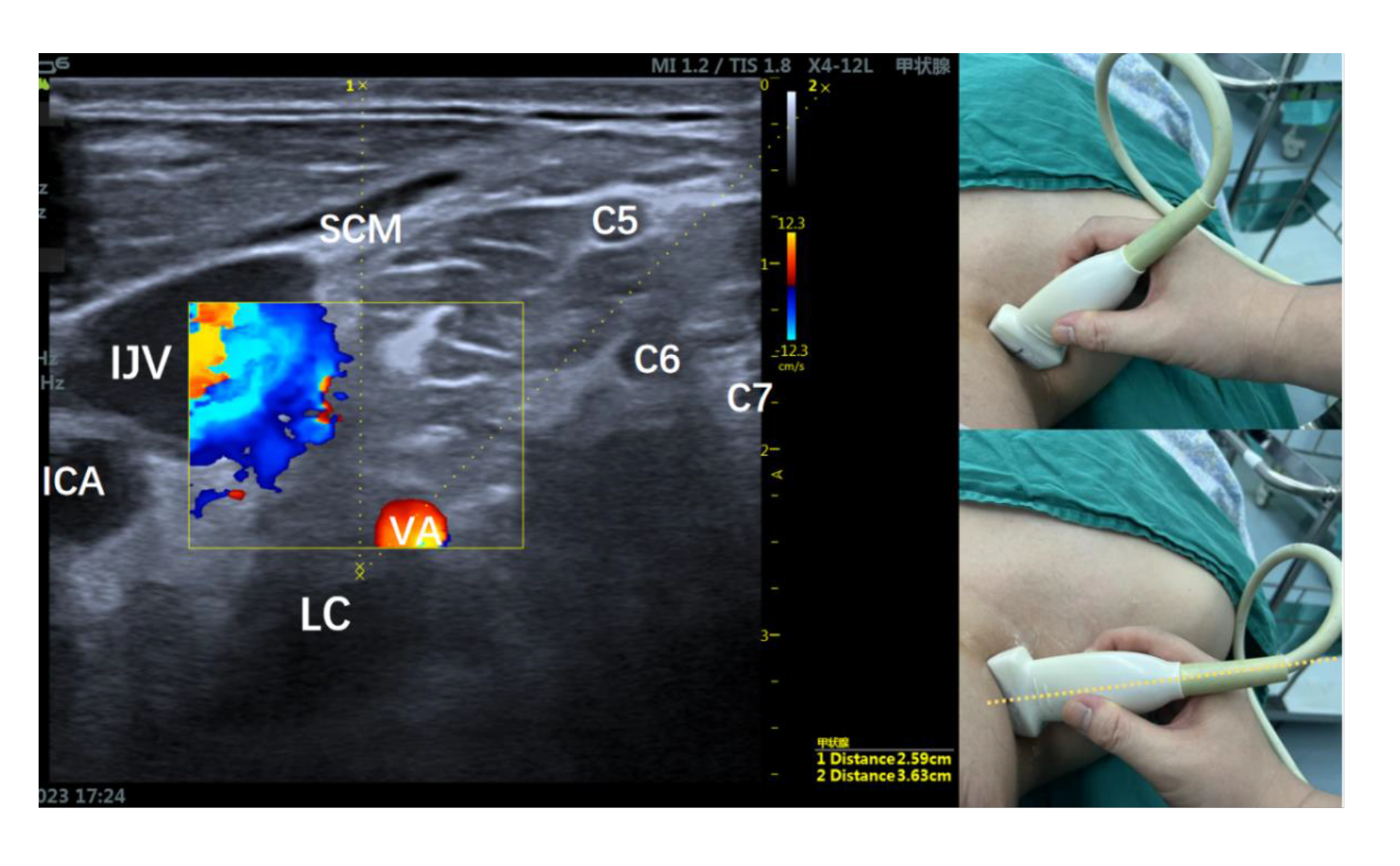

Supplement: Supplementary Figure 1 — Ultrasonographic image of the needle paths for stellate ganglion block using the lateral paravein out-of-plane approach. The yellow dashed line in the actual diagram shows the out-of-plane and in-plane approaches to the internal jugular vein, corresponding to Distance 1 and Distance 2 in the ultrasound image, respectively. [file Image_1.TIF]
